# Palato-pharyngo-laryngeal myoclonus with recurrent retrograde feeding tube migration after cerebellar hemorrhagic stroke: a case report and review of hypertrophic olivary degeneration

**DOI:** 10.1186/s12883-020-01800-6

**Published:** 2020-06-03

**Authors:** Jamie L. Fleet, Ronelle Calver, Gihan C. Perera, Zhihui Deng

**Affiliations:** Division of Physical Medicine and Rehabilitation, Department of Medicine, McMaster University, Hamilton, Canada

**Keywords:** Symptomatic palatal myoclonus, Hypertrophic olivary degeneration, Guillain-Mollaret triangle

## Abstract

**Background:**

Palato-pharyngo-laryngeal myoclonus, a variant of palatal myoclonus, is characterized by involuntary rhythmic movements of palatal, pharyngeal, and laryngeal muscles. Symptomatic palatal myoclonus is classically associated with hypertrophic olivary degeneration on MRI imaging due to a lesion in the triangle of Guillain-Mollaret.

**Case presentation:**

We report a case of palato-pharyngo-laryngeal myoclonus in a patient post-cerebellar hemorrhagic stroke who presented with recurrent retrograde migration of his gastrojejunostomy feeding tubes. Treatment with either divalproex sodium or gabapentin resulted in a significant decrease in his gastrointestinal symptoms and no further episodes of gastrojejunostomy tube migration.

**Conclusions:**

This case study indicates that the movement disorder associated with hypertrophic olivary degeneration may involve the gastrointestinal system. Anticonvulsants, such as gabapentin and divalproex sodium, may reduce the severity of gastrointestinal symptoms in cases associated with hypertrophic olivary degeneration. The anatomy of the Guillain-Mollaret triangle and the pathophysiology of hypertrophic olivary degeneration are reviewed.

## Background

Palatal myoclonus, also known as palatal tremor, is a rare condition first described by Politzer in the 1860’s characterized by involuntary persistent rhythmic muscle contraction involving the soft palate [1]. Two types of palatal myoclonus have been described: a symptomatic form attributable to a pathologic lesion involving the brainstem or cerebellum often identified on MRI imaging, and an essential form without an identifiable structural cause [2]. Palato-pharyngo-laryngeal myoclonus is a rare variant of palatal myoclonus affecting not only the palate but the pharynx and larynx as well, which may result in dysphagia, dysphonia, and dysarthria [3–5]. Upon review of the current literature, there are minimal, if any, gastrointestinal (GI) symptoms reported by patients with this condition. We report a novel case of recurrent retrograde migration of enteral feeding tubes associated with palato-pharyngo-laryngeal myoclonus following a cerebellar hemorrhagic stroke.

## Case presentation

A 57-year-old man was admitted to our acquired brain injury (ABI) rehabilitation unit 8 months after he suffered a large right cerebellar hemorrhage with significant mass effect requiring an emergent posterior fossa decompressive craniectomy. His past medical history included hypertension, vertigo, a remote concussion, and history of a fractured jaw. He developed significant dysphagia early following his stroke, including difficulty with secretion management. The dysphagia was likely multifactorial, related to the cerebellar bleed with initial mass effect and possible subsequent structural injury to the brain stem (central) and cranial nerves (peripheral) as well as an early altered level of consciousness (drowsiness, somnolence) following his stroke. A percutaneous endoscopic gastrostomy (PEG) tube was placed 11 days after his stroke, but refractory vomiting and aspiration pneumonia eventually necessitated gastrojejunostomy (GJ) tube placement. He continued to have ongoing issues with vomiting despite a trial of metoclopramide. Imaging performed 1 month after placement of the GJ tube revealed retrograde migration with the tip of the catheter visualized within the oropharynx, which was approximately 14 weeks after his stroke. The GJ tube was then replaced under fluoroscopic guidance. Within 2 weeks the tube had again migrated and was visualized within the esophagus on imaging. He underwent endoscopic placement of a gastrostomy tube with addition of a jejunal extension. Ten weeks after placement, the GJ tube had migrated with the tip now located in the duodenum. At this point, his chronic vomiting appeared to be improving with a trial of erythromycin so no further adjustments to the tube were made.

Upon admission to our ABI unit he had ongoing dysphagia, significant hypophonia, and intermittent episodes of regurgitation and vomiting, though often only small amounts of bile or fluids. The vomiting was not stereotyped, nor did he have long episodes where he was completely symptom free, as seen in cyclic vomiting syndrome [6]. He had two further episodes of retrograde tube migration requiring replacement by interventional radiology. A video fluoroscopy study was performed 10 months after his stroke that revealed rhythmic pulsations of the palate, pharynx, and larynx involving the vocal cords, in keeping with palato-pharyngo-laryngeal myoclonus (see video). Subsequently, a brain MRI was completed showing apparent unilateral hypertrophy and increased FLAIR signal in the left inferior olive suggestive of hypertrophic olivary degeneration.

Several medication trials including clonazepam, divalproex sodium, and gabapentin, were undertaken to help control his palato-pharyngo-laryngeal myoclonus. Medications were administered independently with no combination therapies trialed. Clonazepam was poorly tolerated due to drowsiness and thus discontinued. Divalproex sodium titrated up to 1000 mg per day and gabapentin titrated up to 600 mg per day did not lead to significant changes in the frequency or intensity of his myoclonus on repeat video fluoroscopic studies. However, his GI symptoms improved significantly with these medication trials such that he no longer vomited and there were no further incidents of retrograde migration of the feeding tube. Imaging performed 3 months after the initiation of the medication trials and 2 months after the most recent GJ tube replacement demonstrated the tip of the tube appropriately located within the jejunum.

## Discussion and conclusions

**Fig. 1:**
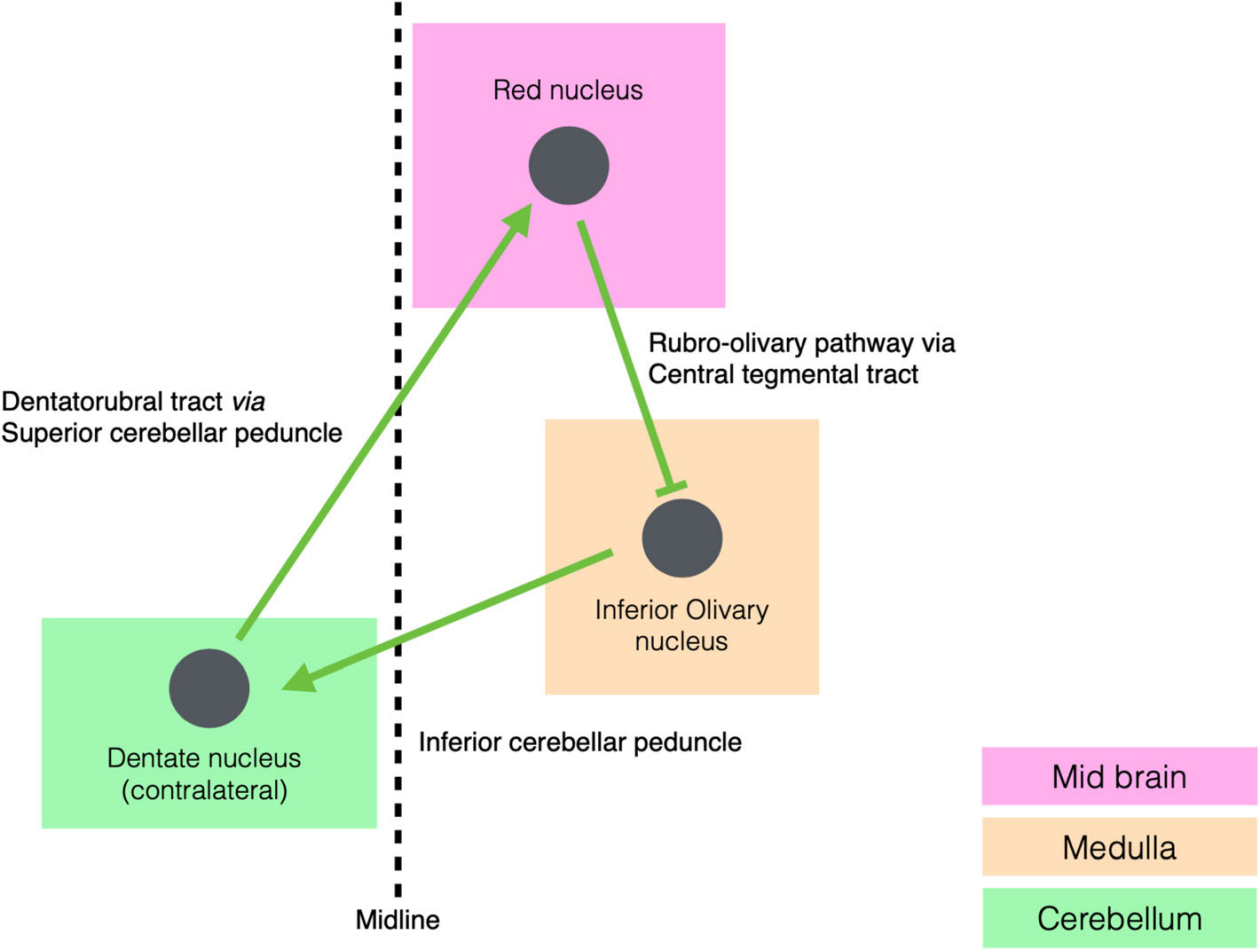
Guillain-Mollaret Triangle. Graphical representation of The Guillain-Mollaret triangle (dentate-rubro-olivary pathway) with connections between the dentate nucleus, contralateral red nucleus, and inferior olivary nucleus highlighted

Symptomatic palatal myoclonus is attributable to hypertrophic olivary degeneration (HOD), a transsynaptic degeneration caused by a lesion in the anatomic triangle of Guillain-Mollaret [7, 8]. The Guillain-Mollaret triangle, also known as the dentato-rubro-olivary pathway, is a neural circuit involved in motor modulation. The triangle comprises the ipsilateral red nucleus in the midbrain, inferior olivary nucleus (ION) in the medulla, and the contralateral dentate nucleus in the cerebellum (Fig. 1). The efferent fibres from the dentate nucleus ascend through the superior cerebellar peduncle, decussate at the midline, and then connect with the contralateral red nucleus in the rostral midbrain. The efferent fibers of the red nucleus then descend via the central tegmental tract and reach the ipsilateral inferior olivary nucleus. The inferior olivary nucleus completes the triangle by sending decussating efferent fibers via the inferior cerebellar peduncle to the original dentate nucleus within the cerebellum [7, 9].

The exact mechanism underlying palatal myoclonus is not completely understood. The inferior olivary neurons are electronically coupled via gap junctions and show spontaneous rhythmic electric discharges in animal models [10]. The dentate nucleus primarily inhibits the inferior olivary nucleus via GABA-mediated modulation. When a pathologic lesion interrupts this inhibitory modulation, inferior olivary neurons enlarge and develop abnormal soma-somatic gap junctions within weeks to months [11]. It has been established that HOD occurs with lesions involving the dentato-rubral pathway in the superior cerebellar peduncle or the rubro-olivary pathway in the central tegmental tract, but not in nearby areas [9]. This supports the theory that it is the loss of inhibitory input from the dentate nucleus to the ION that causes overactivity in the latter [11]. As a result, the ION may become an autonomous oscillator due to its intrinsic rhythmic capabilities leading to hyperkinetic movement disorders [11]. Symptoms can present anywhere from 1 month to 8 years after lesion to the Guillain-Mollaret triangle, though most commonly onset is within the first year [9, 12–15].

In addition to the hypothesis of the ION as an oscillator in the pathophysiology of palatal myoclonus, a second mechanism has been proposed that maladaptive cerebellar plasticity may facilitate and amplify the rhythmic output from the hypertrophic ION [11]. Moreover, a recent case report of oculopalatal myoclonus following medullary infarcts without development of HOD suggests a possible source of oscillation outside the ION [16].

The nucleus ambiguus (found in close proximity to the ION) innervates the pharyngeal, laryngeal, and palatal musculature as well as upper esophagus via cranial nerves 9,10, and 11 [11, 17]. Interestingly, HOD commonly results in palatal myoclonus caused by the rhythmic contraction of the levator veli palatini muscle [18]. It is unclear why this specific muscle is most often affected. Sometimes other muscles innervated by the nucleus ambiguus may be involved leading to palato-pharyngo-laryngeal myoclonus. Connections from the central tegmental tract to the nucleus ambiguus have been implicated as part of the pathway leading to palatal myoclonus [9, 19]. Possibly the close proximity of the two structures in the medulla (ION and nucleus ambiguus) may also play a role in this relationship.

Other areas of body, such as the extraocular muscles, head, diaphragm, and even extremities, can also be affected in HOD [18]. The remote effects of HOD have been demonstrated with EMG studies that revealed rhythmic electrical discharges in leg musculature, though patients did not necessarily present with overt myoclonus in their extremities [20]. Whether HOD can have an effect on autonomic function, such as in the GI tract, is unknown.

Consistent with previously documented findings in HOD, our patient presented with myoclonus involving the palate, pharynx, and larynx causing hypophonia and contributing to his multifactorial dysphagia. He also presented with disrupted upper GI motility, likely retrograde peristalsis, causing recurrent GJ tube migration – an extremely rare clinical phenomenon. Retrograde GJ tube migration into the upper GI tract has been described secondary to persistent and forceful vomiting [21]. The patient in this case did not have forceful vomiting, and his emesis was generally small amounts of bile and rarely his enteral feeds. We hypothesized that his GI symptoms may be related to his HOD given the delayed onset post-stroke; his nausea and vomiting were first documented 3 weeks after his cerebellar hemorrhage and the first occurrence of enteral feeding tube migration was at 14 weeks.

**Fig. 2:**
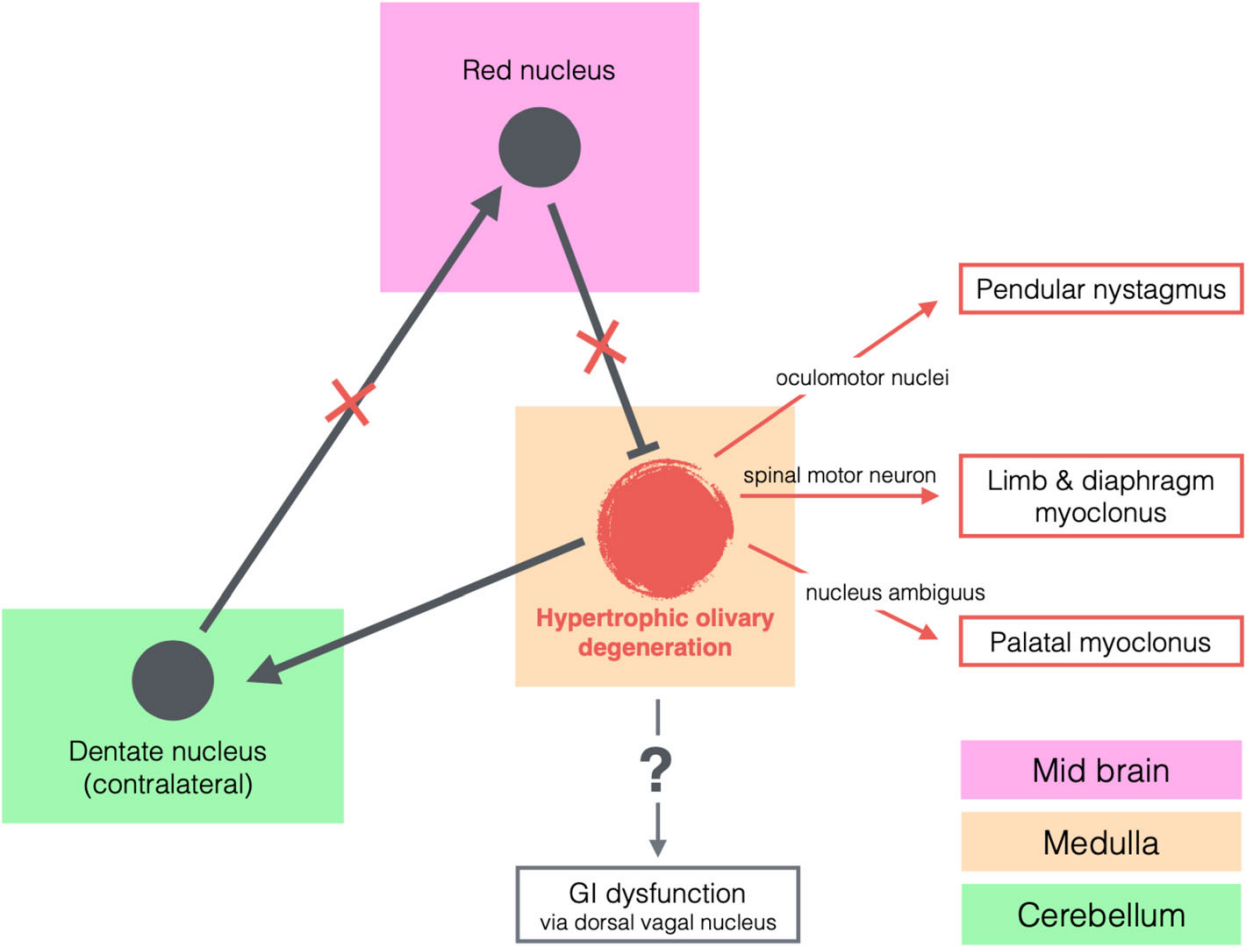
Hypertrophic Olivary Degeneration Pathway. Proposed mechanism for pathologic signs developed in the setting of hypertrophic olivary degeneration. With loss of inhibitory input to the inferior olivary nucleus, hypertrophic olivary degeneration occurs. Anatomic structures affected by hypertrophic olivary degeneration are identified by text with arrows leading to resultant movement disorders

The GI tract has both intrinsic enteric neural plexuses and extrinsic innervation from parasympathetic and sympathetic pathways. While the sympathetic system inhibits motor activity, the parasympathetic system modulates the tone and motility of GI musculature via the vagal nerve. Vagal innervation of the GI tract arises from the dorsal vagal nucleus in the medulla. As discussed above, the nucleus ambiguus is often affected in HOD leading to palatal myoclonus. Given the remote effects of HOD detailed in previous literature, other motor nuclei in the brainstem (for example, innervating eye movements) and motoneurons in the spinal cord (for example, innervating the diaphragm and extremities) may also be affected. In this case, we postulate that the dorsal vagal nucleus may have been affected by HOD resulting in this patient’s atypical GI symptoms (retrograde peristalsis) in addition to his palatal myoclonus (Fig. 2). Alternatively, but less likely, this may be related to the close proximity of the efferent fibres of the dorsal vagal nucleus and the nucleus ambiguus via the vagal nerve. Another possibility is that the nucleus ambiguus itself may play a role in our patient’s GI dysfunction, as the gastric innervation by the nucleus ambiguus has been demonstrated in animal models [22]. Further research in this area is warranted.

There is no widely accepted pharmacologic treatment for symptomatic palatal myoclonus, though there have been case reports of successful treatment with valproic acid or other anticonvulsants [23, 24], trihexyphenidyl [25], and piracetam [26]. Gabapentin and memantine were observed to be effective in treating some patients with pendular nystagmus secondary to HOD [12]. Based on our theory that his GI symptoms were related to his HOD and palatal myoclonus, several medication trials were initiated in our patient. While his palato-pharyngo-laryngeal myoclonus did not show significant response to treatment with divalproex sodium or gabapentin, his GI symptoms dramatically improved, even with each medication used singly. This may suggest that the retrograde tube migration was more related to the HOD effect on the dorsal vagal nucleus rather than the palato-pharyngo-laryngeal myoclonus itself. Divalproex sodium was effective in improving his GI symptoms, however the patient’s family was concerned about the side effect profile, therefore it was weaned down and switched to gabapentin. Prior to initiation of these medication trials, his GJ tube required replacement on four occasions within 7 months. Additionally, while his vomiting was unresponsive to more traditional anti-emetics, such as metoclopramide, it did improve with addition of anticonvulsants. At discharge, he was on gabapentin 600 mg PO QHS with no significant vomiting or migration of his GJ tube within the previous 2 months, lending support to his GI symptoms being related to underlying HOD.

## Conclusion

This is the first case report of significant GI symptoms associated with HOD following a lesion in the Guillain-Mollaret triangle. If patients with HOD present with atypical GI symptoms unresponsive to standard treatments, anticonvulsants, such as divalproex sodium and gabapentin, may be effective. Further studies are needed to determine the underlying pathophysiology of HOD and associated movement disorders in order to better target clinical treatment.

## Supplementary information

**Additional file 1.** Top down view of pharynx and larynx demonstrating rhythmic pulsations, most prominent on the left vocal cord.

## Data Availability

Not applicable.

## Abbreviations

ABI: Acquired brain injury
GI: Gastrointestinal
GJ: Gastrojejunostomy
HOD: Hypertrophic olivary degeneration
ION: Inferior olivary nucleus
